# The Population Decline and Extinction of Darwin’s Frogs

**DOI:** 10.1371/journal.pone.0066957

**Published:** 2013-06-12

**Authors:** Claudio Soto-Azat, Andrés Valenzuela-Sánchez, Ben Collen, J. Marcus Rowcliffe, Alberto Veloso, Andrew A. Cunningham

**Affiliations:** 1 Laboratorio de Salud de Ecosistemas, Facultad de Ecología y Recursos Naturales, Universidad Andres Bello, Republica 252, Santiago, Chile; 2 Institute of Zoology, Zoological Society of London, Regent’s Park, London, United Kingdom; 3 Centre for Biodiversity & Environmental Research, University College London, London, United Kingdom; 4 Departamento de Ciencias Ecológicas, Facultad de Ciencias, Universidad de Chile, Las Palmeras, Nuñoa, Chile; Smithsonian’s National Zoological Park, United States of America

## Abstract

Darwin’s frogs (*Rhinoderma darwinii* and *R. rufum*) are two species of mouth-brooding frogs from Chile and Argentina. Here, we present evidence on the extent of declines, current distribution and conservation status of *Rhinoderma* spp.; including information on abundance, habitat and threats to extant Darwin’s frog populations. All known archived *Rhinoderma* specimens were examined in museums in North America, Europe and South America. Extensive surveys were carried out throughout the historical ranges of *R. rufum* and *R. darwinii* from 2008 to 2012. Literature review and location data of 2,244 archived specimens were used to develop historical distribution maps for *Rhinoderma* spp. Based on records of sightings, optimal linear estimation was used to estimate whether *R. rufum* can be considered extinct. No extant *R. rufum* was found and our modelling inferred that this species became extinct in 1982 (95% CI, 1980–2000). *Rhinoderma darwinii* was found in 36 sites. All populations were within native forest and abundance was highest in Chiloé Island, when compared with Coast, Andes and South populations. Estimated population size and density (five populations) averaged 33.2 frogs/population (range, 10.2–56.3) and 14.9 frogs/100 m^2^ (range, 5.3–74.1), respectively. Our results provide further evidence that *R. rufum* is extinct and indicate that *R. darwinii* has declined to a much greater degree than previously recognised. Although this species can still be found across a large part of its historical range, remaining populations are small and severely fragmented. Conservation efforts for *R. darwinii* should be stepped up and the species re-classified as Endangered.

## Introduction

There are two species of Darwin’s frogs: the Northern Darwin’s frog (*Rhinoderma rufum*) and the Southern Darwin’s frog (*R. darwinii*), both of which inhabit temperate forests in central and south Chile and, in the case of the latter, also in adjacent areas of Argentina [1], [2]. *Rhinoderma darwinii* was named in honour of Charles Darwin [3], who first found this frog on December 1834, on the Island of Lemuy, Chiloé Archipelago [4]. *Rhinoderma rufum* was originally described in 1902 [5], but after some debate (for some time it was considered a form of *R. darwinii*) [6], [7], it was confirmed as a separate species in 1975 [8]. With the snout-vent length of adults ranging from 2.2 to 3.2 cm [6], [8], [9], [10], Darwin’s frogs have a fascinating method of parental care that sets these frogs apart from all other known amphibians (7,044 spp.) [11]. Males care for their young by incubating them in their vocal sacs for part of their development, a process first documented by Jiménez de la Espada [12] and since termed neomelia [6], [8], [13], [14], [15], [16]. Along with seahorses (genus *Hippocampus*), *Rhinoderma* spp. are the only known living vertebrates where males incorporate developing embryos into a specialized sac, giving the appearance of being “pregnant”. The two *Rhinoderma* spp. differ in their expression of this reproductive behaviour: while *R. rufum* expels larvae into water bodies prior to metamorphosis, *R. darwinii* males do not release the young until they have metamorphosed [17]. The two species can also be distinguished morphologically: *R. rufum* has a well-developed, transparent interdigital membrane between all five toes of the hind feet, a prominent metatarsal external tubercle and a diffuse pattern of white marks on a black background on the ventral surface of the body. Whereas in *R. darwinii*, the interdigital membrane is thicker, but present only between hind toes III–IV and IV–V; the metatarsal tubercle is smaller; and the white ventral markings are generally larger and extend farther caudally to include the hind feet [8], [18]. Other differences include: smaller intra-vocal sac larvae of *R. darwinii*, compared to the larger larvae of *R. rufum* [14], [19]; differences in call patterns [8], [20]; and characteristic karyotypes [21].

*Rhinoderma rufum* has not been recorded since 1980 [20], [22] while *R. darwinii* is no longer found at some locations from which the species was recently abundant [23]. The reasons for these apparent disappearances remain poorly understood. Throughout the historical distribution of *R. rufum*, and within the northern range of *R. darwinii*, there has been extensive habitat degradation, mainly due to the large-scale replacement of native forest with pine (*Pinus radiata*) and eucalypt (*Eucalyptus globulus*) plantations [1], [2], [24], [25]. Habitat loss, however, does not fully explain the enigmatic disappearances of *R. rufum* from its entire historical range or of the declines of *R. darwinii* from wild protected areas (WPAs), such as National Parks and other undisturbed ecosystems.

*Rhinoderma rufum* is classified as Critically Endangered by the IUCN [2], and is ranked #45 on the amphibian evolutionarily distinct and globally endangered (EDGE) list [26], while *R. darwinii* is listed as Vulnerable [1], but there is little information on their current distributions or abundances. Such information is required in order to develop adequate conservation strategies for these species. Here, we present evidence on the extent of the declines, current distribution and conservation status of *Rhinoderma* spp., including information on the relative abundance, population size, population density and habitat of, and threats to, extant *R. darwinii* populations.

## Materials and Methods

### Ethics Statement

This study was carried out in strict accordance with the recommendations in the guidelines for use of live amphibians and reptiles in field research compiled by the American Society of Ichthyologists and Herpetologists (ASIH). Research was approved by the ZSL Ethics Committee and was conducted following Chilean and Argentinian wildlife regulations and according to permits 1241/08, 7377/09, 7993/10 and 300/12 of the Livestock and Agriculture Service (SAG) and 20/09, XI-01/09, 28/11 and X-03/11 of the National Forestry Corporation (CONAF) both in Chile, and permit 1119/11 of the National Parks Administration (APN) in Argentina. Archived amphibians were examined in their museum of origin, by the authors or museum staff, with specific permission given by all 50 zoological institutions specified in Appendix S1.

### Historical Distribution

A review of the scientific literature and of museum records was conducted to establish the historical distributions of *R. darwinii* and *R. rufum*. Museums known to us to contain *Rhinoderma* spp. in their collections were either visited or contacted to obtain information on the dates and locations from which their specimens had been collected. The largest collections in Europe and South America were visited and each metamorphosed specimen in these collections was examined to confirm the species identity. From the museums not visited, herpetologists in charge of the collections examined the individuals, and photographs of specimens of uncertain identification were examined by us to confirm the species identity. Using these data, historical distribution range maps were created for each *Rhinoderma* spp. following the α-minimum convex polygon method [27].

### Current Distribution

Extensive surveys throughout the historical distribution of *R. rufum* and *R. darwinii* in Chile and Argentina were carried out from October 2008 to March 2012. Directed surveys were designed based on the results of the historical distribution and were conducted at locations where the presence of *Rhinoderma* spp. had been identified in publications or museum records. In addition, an awareness-raising campaign throughout the historical distributions of *Rhinoderma* spp. (and covering all habitat types with the ranges) was conducted with the use of informative leaflets, presentations and interviews with local people and park rangers. This allowed coverage of large areas to help identify current presence/absence of Darwińs frogs. Also, a small number of sightings during the period of this study were obtained from reliable sources (knowledgeable herpetologists or photographic proof) for sites that could not be visited.

Darwin’s frogs are diurnal [9], therefore surveys were conducted during daylight hours. In order to maximise the likelihood of finding frogs, all surveys were conducted between October and March, when the frogs are reproductively active and call more frequently [8]. Accessible areas at each visited location were carefully searched by 2–6 herpetologists for visual and auditory encounters with *Rhinoderma* spp. Absence of *Rhinoderma* spp. in a site was determined after completing a minimum of two visits carried out in different years, each search effort of no less than 5 hours duration.

### Estimated Date of Extinction

For *R. rufum*, all years for which records of historical sightings had been recorded in museum archives and in the scientific literature, were obtained. Following Solow [28], these were analysed using the non-parametric method, optimal linear estimation (OLE), within the statistical package, R (v. 2.13.1), to test the null hypothesis that the species is extant.

### Habitat

For each population of Darwin’s frog found, the vegetation coverage of the site was characterised as: a) forest; b) shrub and bushes; and c) grassland, moss and coarse woody debris (CWD). Temperature and relative humidity were measured at 1–2 cm above the ground at each site visited. For those areas with historical presence of *Rhinoderma* spp., but no evidence of an extant population, current type of land use and presence of other amphibians were recorded. The degree of habitat perturbation was categorised as none, low, high or severe, according to the status of protection of the land, proximity to humans, and/or exploitation of the immediate forest.

### Abundance

It is known *R. darwinii* generally form colonies with high site fidelity in small areas (e.g. a clearing in the forest) [9]. At locations where Darwin’s frogs were found, sites were delimited and a standardised search effort of one hour by two researchers was conducted in order to obtain information on relative abundance. Searches were done in such a manner that survey effort was equal throughout each site. Captured frogs were temporarily removed, safely contained in individual sealed plastic bags and put back immediately after the capture session in the exact place of capture. Each frog was handled using new, disposable nitrile gloves (prior to release, morphometric data and non-invasive skin swabs for complementary studies were obtained). This procedure was conducted for at least two visits for each population in order to estimate a relative abundance index (RAI), which was calculated for each population as follows: RAI = C×F^−1^, where C is the sum of counts (captures) and F is the frequency of capture sessions at each population [29], [30], [31]. Populations were geographically classified as Coastal, Andes, Chiloé Island and South. Statistical analyses were performed using SPSS (v. 20.0) to detect any significant difference in relative abundance between geographical groupings and between different degrees of human impact.

**Figure 1:**
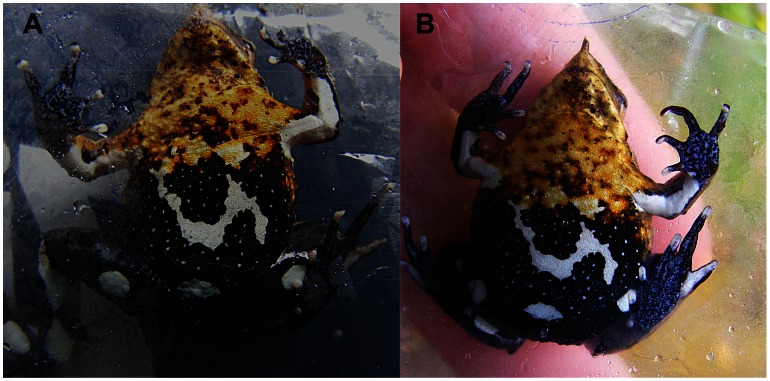
Individual ventral pattern in Darwin’s frog. Recaptured Southern Darwin’s frog (*Rhinoderma darwinii*). A) 25 November 2009, and B) 8 January 2011.

For those populations with at least four consecutive standardised capture sessions (1 hour by two researchers per session), each session separated by 24 hours, capture histories were created through a modified non-invasive capture-mark-recapture (CMR) technique, based on the individually-unique black-and-white coloration patterns of the ventral body recorded in each captured *R. darwinii* (Figure 1). The selection of these populations was based on three criteria: 1) representation of all four geographic regions within the distribution range of *R. darwinii*; 2) different degrees in *R. darwinii* abundance, previously obtained by RAI values; and 3) accessibility, i.e. closeness to a road or village. Simple, behaviour, time and heterogeneity models, and mixtures of them, were performed using the closed captures Huggins full heterogeneity function in programme MARK (v. 6.1) to estimate population sizes [32]. The area of the site containing each population was measured in order to calculate population densities.

## Results

### Historical Distribution

A total of 2,244 *Rhinoderma* specimens from 50 institutions were examined. Based on anatomical features; 789 of the specimens which had been catalogued as *R. darwinii* were re-classified as *R. rufum*. As a consequence, a total of 1,226 *R. darwinii* and 1,018 *R. rufum* were identified (see Appendix S1 in supplementary data).

**Figure 2:**
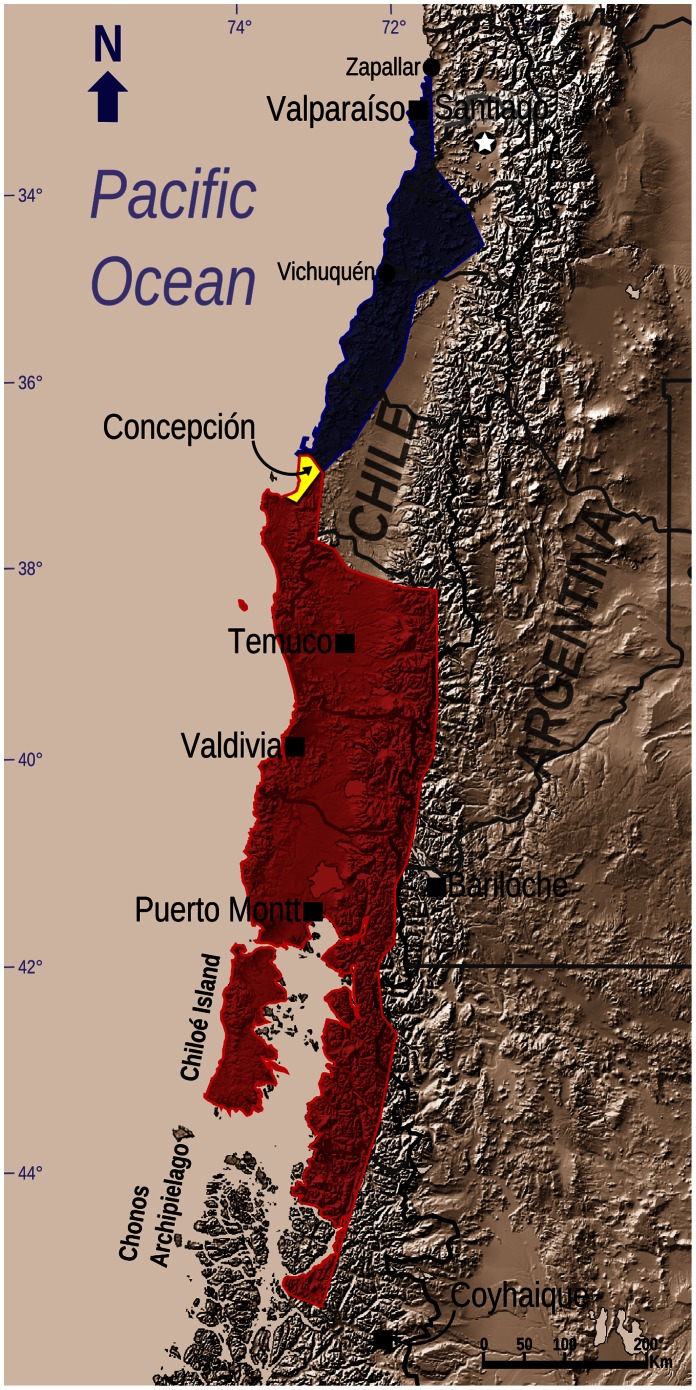
Historical distribution range map for Darwin’s frogs. Blue, Northern Darwin’s frog (*Rhinoderma rufum*); red, Southern Darwin’s frog (*Rhinoderma darwinii*); yellow, area of sympatry.

Thirteen unique locations for *R. rufum* were reported in the scientific literature [5], [8], [13], [20], [33], [34], [35], seven additional unique locations were identified from the collection data for archived specimens, and a further location was provided by G. Medina-Vogel, pers. comm. (see Appendix S2). For *R. darwinii*, 24 unique locations were reported in the scientific literature [4], [6], [9], [13], [16], [23], [36], [37], [38] and an additional 90 unique locations were established from the collection data for archived specimens. Based on these results, and the new locations of *R. darwinii* found in the current study (see below), the historical ranges for *R. rufum* and *R. darwinii* are presented in Figure 2.

### Current Distribution

**Figure 3:**
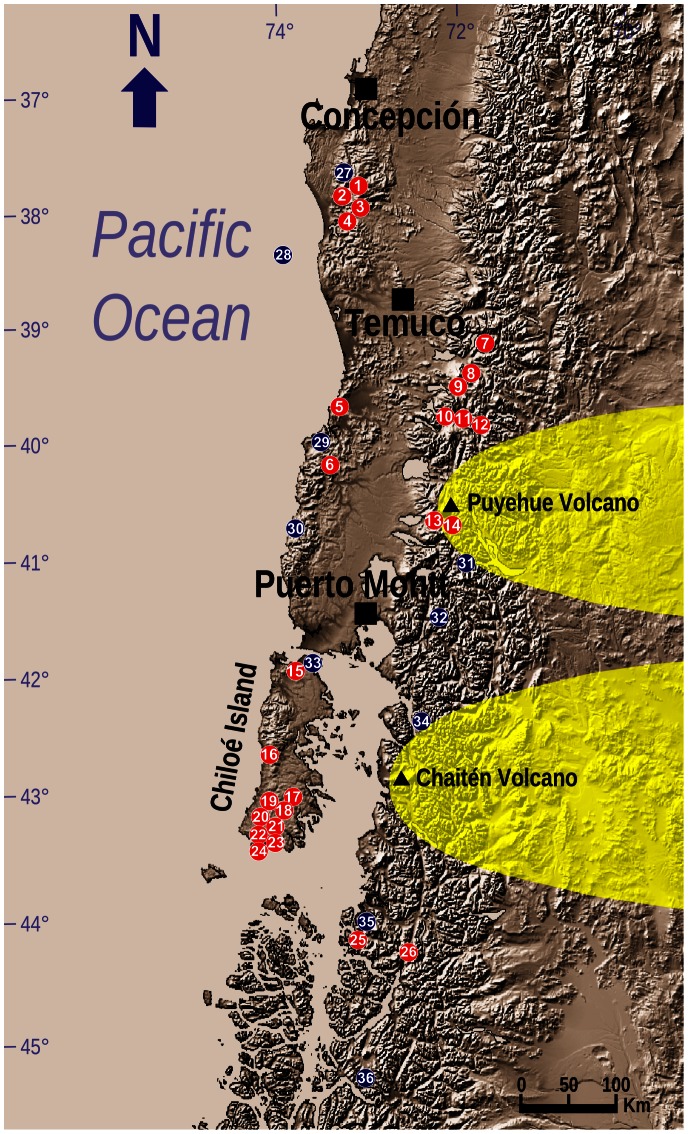
Extant populations of the Southern Darwin’s frog (*Rhinoderma darwinii*) in south Chile and Argentina. Red circles, studied populations; blue circles, species identified, but population status uncertain; black triangles and yellow areas, recent volcanic eruptions and their areas of direct influence.

A total of 223 sites were surveyed for Darwin’s frogs: 46 within the historical range of only *R. rufum*, 157 within the historical range of only *R. darwinii*, and 20 sites within the area of sympatry (Figure 2). Despite the extensive search effort and surveys of every recorded location of the species, no individuals of *R. rufum* were either observed or heard. For *R. darwinii*, however, we found 26 areas (Figure 3) with extant frogs within which the species was present in a total of 36 sites. Generally these areas were extremely isolated from each other, except for populations in southern Chiloé Island (Figure 3). *Rhinoderma darwinii* sites >2 km from each other were determined to be separate populations (and therefore different areas), whilst sites <2 km apart were considered to be subpopulations (and therefore sites). This distinction was based on studies of other amphibians with poor dispersal abilities [39], [40], as is the case for *Rhinoderma* spp [9]. To facilitate our abundance analyses, however, we treated each of the 36 *R. darwinii* sites as separate “populations”. In 10 additional areas, the presence of the species was confirmed either by the detection of a single *R. darwinii* (Cochamó and Caulín), or through obtaining reliable information about the species’ presence: Cayucupil, Isla Mocha, Chaihuín, Hueicolla, Huinay, Río Marchant and Río Cuervo in Chile; and Puerto Blest in Argentina (Figure 3). These 10 areas were not included in our abundance analysis as the population status was uncertain.

### Estimated Date of Extinction

Years of sightings for *R. rufum* are detailed in Appendix S2. Considering the five most recent sightings [41], [42] the mathematical model we used inferred the date of extinction of *R. rufum* as 1982 (95% CI, 1980–2000).

### Habitat

Details of the habitat and identified threats at specific locations surveyed for *Rhinoderma* spp. at which they were known to have been present but were not found during the current study are presented in Table 1. Briefly, 20 of 24 such sites were classified as having moderate to severe anthropogenic habitat perturbation, while four sites (1 *R. rufum* site, 3 *R. darwinii* sites) were identified as having no or low anthropogenic habitat perturbation. One of these last sites (Amarillo), however, has suffered from volcanic activity, which has been associated with the recent disappearance of *R. darwinii* (C.S-A. & A.V-S., personal observations).

**Table 1:** Characteristics of known locations of Darwin’s frogs (*Rhinoderma darwinii* and *Rhinoderma rufum*) from which the species apparently disappeared during or since the 20^th^ Century.

Location	Species^a^	Last year sighted	Historical abundance^b^	Source	Human habitat perturbation		
					Degree^c^	Possible causes of decline^d^	Other amphibians^e^
Zapallar	RR	1966	low	Formas et al. 1975	++	U/T	AN/PT
Los Quillayes	RR	1908	low	Barros 1918	+	A	no
Nilahue	RR	1908–12	low	Barros 1918	++	A	RA/PT
Paredones	RR	1977	low	CIZ 112	+++	U/A	no
La Barranca Alta	RR	1951	low	Formas et al. 1975	++	A/F	BT
Cutemu	RR	1908–12	low	Barros 1918	++	U/A/F	no
Lago Vichuquén	RR	1969	medium	MNHN 1978.253	++	U/T/A/F	PT
Ranguilí	RR	1912	low	Barros 1918	++	A	RA
Hualañé	RR	1908	low	Barros 1918	++	U/A	XL
Constitución	RR	1917–27	low	Wilhelm 1927	++	F	BT/PT
Río Longaví	RR	1975	low	G. Medina-Vogel pers. comm.	++	U/A	CG/PT
Nueva Aldea	RR	1938	high	ZMH A10975–95	+++	U/A/F	no
Cerro Caracol	RR/RD	1965	high	MZUC 011848/024832	+++	U/F	ER/PT
Chiguayante	RR/RD	1979	high	FMNH 209292–391/211144–209	++	U/F/E	ER/PT
Hualqui	RR/RD	1977	low	FMNH 211071	++	A/T/F	ER/PT
San Pedro	RR/RD	1980	medium	CIZ 412–5/502	++	U/F	ER
Arauco	RR/RD	1904	low	BMNH 1904.10.26.109–10	+++	U/A	PT
Ramadillas	RR/RD	1971	low	MZUC 11642	++	U/A/F	BT/PT
Nahuelbuta NP	RD	2006	high	M. Higuera pers. com.	–	E	AB/EN
Lago Lanalhue	RD	1963	low	MZUC 011851/024818	++	U/T/A/F	BT/EC/PT
Cerro Ñielol	RD	1978	high	Rageot 1978.	+	U/T	no
La Saval	RD	1978	high	CIZ 271-3	++	U/T	CG/PT
Huachocopihue	RD	1967	high	Formas et al. 1969	++	U	BT
Amarillo	RD	2008	high	Soto-Azat pers. obs.	–	V	BA

a RR = Rhinoderma rufum, RD = Rhinoderma darwinii.

b Based on number of archived specimens found, collected during a single session. Low = 1 to 5, medium = 6 to 10, high >10.

c – = none, location within a wild protected area (WPA) or undisturbed ecosystem;+ = low, location in a native forest exploited for firewood or near a trail frequently transited within a WPA;++ = high, location in a severely exploited native forest, or near a town or development infraestructure; and+++ = severe, location within urban settlements.

d U = urban, T = tourism, A = agriculture, F = forestry, E = extraction of Darwin’s frog, and V = volcanic eruption.

e AB = Alsodes barrioi, AN = Alsodes nodosus, BA = Batrachyla antartandica, BT = Batrachyla taeniata, CG = Calyptocephalella gayi, EC = Eupsophus contulmoensis, EN = Eupsophus nahuelbutensis, ER = Eupsophus roseus, PT = Pleurodema thaul, RA = Rhinella arunco, XL = Xenopus laevis.

A description of the habitat at each site where *R. darwinii* was found is given in Appendix S3. In all cases, Darwin’s frogs were present only within native forest. From the populations of *R. darwinii* observed, only eight of 36 were found outside WPAs: two within native forest exploited for firewood (Butamalal and Alerzales), one within native forest surrounded by pine and eucalypt plantations (RF Contulmo) and five within privately-owned, non-exploited native forest (El Natre 1 & 2, Coñaripe, Melimoyu 1 & 2).

### Abundance

**Figure 4:**
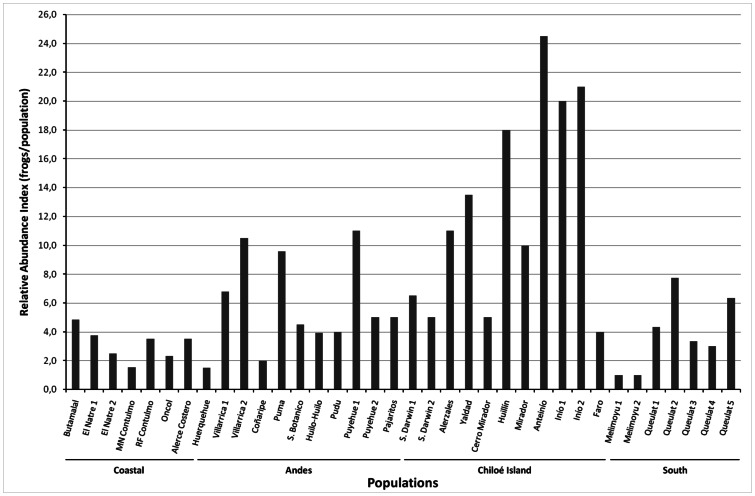
Relative abundance of the Southern Darwin’s frog (*Rhinoderma darwinii*). Frogs/standard search of 1 hour in each of 36 extant populations surveyed.

A total of 648 different *R. darwinii* were captured (120 brooding males, 111 non-brooding males, 218 females and 199 juveniles). Local relative abundances are shown in Figure 4. The RAI values for each *R. darwinii* population gave an average of 7.0 frogs/population (95% CI, 5.0–9.0). Differences in abundance between the four geographical regions were found (one-way ANOVA; *F*_3.32_ = 8.32, *P*<0.001). *Post–hoc* comparisons revealed a higher abundance of frogs in Chiloé when compared with Coast, Andes and South populations (Tukey’s HSD: *P = *0.001, *P* = 0.008, *P* = 0.002 respectively). Differences in abundance according to the degree of human impact were found. Sites with no disturbance showed higher abundance of frogs when compared with sites with anthropogenic disturbance (Mann-Whitney *U*-test; U = 57.0, *P = *0.005).

With CMR data from five populations (El Natre 1, Villarrica 1, Inio 1, Queulat 2 and 3), population size and density averaged 33.2 frogs/population (range, 10.2–56.3) and 14.9 frogs/100 m^2^ (range, 5.3–27.4), respectively. The probability of detection per search was highly variable, ranging from 0.04 to 0.64 across sites, and with evidence for behaviour effects, individual heterogeneity and time-dependent variation in detection probability, at least in the site where search effort was most intense (Inio 1; Table 2).

**Table 2:** Estimated population sizes, calculated using Huggins closed population models and densities of five populations of the Southern Darwin’s frog (*Rhinoderma darwinii*).

Population	*C* a	Model^b^	*p* c	95% CI	Population size (frogs)	95% CI	Population density(frogs/100 m^2^)	95% CI
El Natre 1	4	{b}	0.64	0.33–0.86	10.2	10.1–13.4	5.3	5.3–7.0
Villarrica 1	4	{t}	t_1_∶0.34	0.15–0.59	47.3	32.3–93.3	13.0	8.9–25.7
			t_2_∶0.13	0.05–0.30				
			t_3_∶0.15	0.06–0.33				
			t_4_∶0.04	0.01–0.17				
Inio 1	16	{bht}	h_1_,t_1_∶0.19	0.11–0.3	56.3	56.0–59.5	19.5	19.4–20.7
			h_1_,t_2_∶0.35	0.21–0.51				
			h_2_,t_1_∶0.17	0.10–0.29				
			h_2_,t_2_∶0.56	0.36–0.74				
			p_1_: 0.53	0.38–0.67				
Queulat 2	4	{.}	0.42	0.29–0.56	19.2	17.5–27.1	9.2	8.4–13.0
Queulat 3	4	{.}	0.13	0.04–0.32	33.1	18.9–89.5	27.4	15.6–74.1

a Number of counts.

b Model selected (Model: letter codes indicate detection probability dependence: t = time; b = behaviour; and h = heterogeneity).

c Detection probability. Recapture probability at Inio 1 showed a pattern of initially low values for the early capture occasions, higher values during the middle of the period, then low values, similar to those at the start. A simpler model with only two time periods (t_1_: early/late and t_2_: mid) was therefore preferred to a fully time varying model. h1 and h2 refer to recapture probabilities for heterogeneity mixtures, and p1 to the estimated proportion of the population in mixture 1.

## Discussion

### Historical Distribution

Using as many published and unpublished (museum) records of *Rhinoderma* spp. as we could obtain, we established the historical range of *R. rufum* and *R. darwinii* (Figure 2). According to our results, the distribution of *R. rufum* was much larger than previously recognized [2], as it has been recorded from the foothills of the Andes in the VI Region [33] as well as from the coastal forests of the V Region of Chile (C. Moreno, pers. comm.) [8]. In contrast, according to our data, the historical range of *R. darwinii* has been overestimated [1], as we were unable to locate any records of the species having been found south of the city of Coyhaique or on the Chonos Archipelago.

### Current Distribution and Extent of Declines

Despite multiple, extensive searches, including all previously-known locations for *R. rufum*, we were not able to locate this species. This is in accordance with many other search efforts that have been made over the past 10 years for this species [2], [15], [18], [22], [43].

Identifying the extinction of a species is problematic [41], [44]. There is reluctance to declare a species extinct because of the large conservation implications involved and so as not to facilitate the Romeo effect (giving up on a species too early) or the Lazarus effect (bringing a species back from extinction) [28]. Using the OLE model developed by Solow [28] the sighting record indicates that this species most likely became extinct in the early 1980s. The OLE method does not take into account multiple sightings in a given location, though assumes that sightings effort never falls to zero in intervening years. We felt that the sparse dataset, which we have collated, would not be suitable for an Ederer-Myers-Mantel test [45], although that alternative method should be considered in the future should additional data become available.

We found extant *R. darwinii* in all four main geographic regions (Coastal, Andes, Chiloé Island and South) within its historical range, but only as small and fragmented populations. The current area of occupancy of *R. darwinii* most likely represents a small fraction of its former range. Our data, and those of Crump [9] and Crump and Veloso [23], indicate that this species has disappeared from, or markedly declined in, multiple locations, including those in which was recently abundant. For instance, in the remote area of Melimoyu, Crump [9] studied 146 individuals in 1998 and 120 in 1999 during fieldwork sessions of 10 and 16 days respectively. Although shorter in time, successive expeditions by the authors to the same area during the reproductive season failed to locate any Darwin’s frogs in 2009 (6 days of searching) and found only two individuals in 2011 (5 days). This represents a drastic population decline over the last 12 years in an area of southern Chile only accessible by sea and characterized by an undisturbed ecosystem with a low human presence (∼57 people, Figure 3).

Archived material and scientific literature have shown that both species of Darwin’s frogs have been abundant, at least at some locations [6], [8], [13], [33]. Charles Darwin himself stated among his notes: “It inhabits thick and gloomy forests, and is excessively common in the forest of Valdivia” [46]. In the area of Chiguayante and Cerro Caracol (Concepción) prior to the 1980s, *Rhinoderma* spp. were commonly heard and seen in house gardens (albethey gardens incorporating native forest). In this context, we documented 838 *R. rufum* specimens deposited in different museums, collected by two wildlife collectors from the same area in Chiguayante over the period 1975–1979. An area with high abundance of *R. darwinii* in the past, but apparent current absence, is the Nahuelbuta National Park. In this protected area and its surroundings, the extraction of *R. darwinii* for the illegal pet trade to the United States and Europe was a common practice until the late 1980s [47]. Over-extraction of Darwin’s frogs could have acted as an extinction driver at some localities.

### Habitat Perturbation

No Darwin’s frogs were found near urban or rural settlements; instead they were always associated with specific conditions within native forest. Furthermore, of the *Rhinoderma* spp. populations that have recently disappeared, 22 of 24 suffered anthropogenic habitat perturbation to differing degrees (Table 1). Chilean temperate forests are being rapidly destroyed to supply the increasing global demand for wood and paper products [48]. Echeverria et al. [24] calculated a reduction of natural forest area in the coastal range of central-south Chile of 67% for the period 1975–2000. By 1993, *P. radiata* reached 1.24 million ha in central-south Chile, becoming the largest pine plantations worldwide [49]. Together with eucalypts, they produce drastic changes in atmospheric/substrate humidity, air temperature, luminosity and wind speed [50]. They also promote erosion in environments previously rich in ground cover and may facilitate the dissemination of invasive species [51]. In addition, the conversion of native forest to agriculture has occurred to a large extent, especially towards central Chile [24], [25]. Other causes of habitat loss, such as urbanization and infrastructure development projects, might also play an important role in the disappearance of *R. rufum*. For example, the land surrounding Lake Vichuquén and Zapallar, has been drastically changed for urban and tourist purposes. Urban sprawl in the Greater Concepción area, with over one million inhabitants, now incorporates areas from which the Darwin’s frog populations of Cerro Caracol, Chiguayante, Hualqui and San Pedro have disappeared.

### Other Threats

Small populations are more prone to extinction from environmental and demographic stochasticity [52]. Although volcanic events could be considered as beneficial over geological time-scales, generally they are considered as catastrophic over human time-scales [53]. Two high magnitude plinian volcanic eruptions in the southern Andes have recently affected *R. darwinii* populations. First, the Chaitén volcano eruption from May 2008 to June 2009 and which produced a total of 4 km^3^ of magma and an ash column up to 22 km high [54], is associated with the disappearance of a *R. darwinii* population located 22 km SW from the crater (Amarillo; Table 1, Figure 3). More recently, from June 2011 to May 2012, the Caulle-Puyehue volcano erupted producing ash columns up to 12 km high and with pyroclastic material accumulating in large amounts in the surrounding area [55]. As a consequence, one of two *R. darwinii* populations known in this area (Pajaritos) has apparently disappeared (C.S-A. & A.V-S., personal observations), although additional surveys are required to confirm this (Figure 3).

In the early 1970s, the African clawed frog (*Xenopus laevis*) was introduced to Chile, apparently near Santiago [56]. Mainly by colonization movements, but also with human assistance, *X. laevis* today inhabits an extensive area of central Chile [57], [58]. We detected the presence of this invasive frog near only one site with an historical presence of *R. rufum* (Hualañé; Table 1), however, this species is unlikely to be found at Darwin’s frogs sites as *X. laevis* generally inhabits open lentic waters and human disturbed environments [57]. *Xenopus laevis* has been associated with the emergence and global spread of the amphibian disease chytridiomycosis, caused by *Batrachochytrium dendrobatidis* (*Bd*) [59], [60]. Recently, *Bd* has been identified in wild populations of *X. laevis* in Chile [61] and chytridiomycosis has been reported as a cause of mortality of *R. darwinii* in captivity [36], [43]. The impacts of *Bd* on sympatric wild amphibians, including Darwin’s frogs, in Chile have not been investigated. Further research is required to investigate if *Bd* has been involved in the decline of Darwin’s frogs, in particular their enigmatic declines in, and disappearances from, protected areas.

Other factors that have been cited as possibly causing amphibian declines elsewhere, such as pollution, UV radiation and climate change [62], have not been assessed for Darwin’s frogs. It is possible, however, that global warming with concomitant changes in, for example, precipitation patterns, might negatively impact terrestrial, high humidity dependent, non-migratory species such as Darwin’s frogs, whilst also favouring the dynamics of emerging pathogens [63], [64], [65], [66].

### Habitat Requirements of *Rhinoderma darwinii*

*Rhinoderma darwinii* is known to inhabit small open areas within native forest [9], however, we also found this species in dense forest, *i.e.* in areas with ≥90% mature forest coverage (Oncol, Puma, Pajaritos and Puyehue 2). Even though *R. darwinii* was found in a great variety of vegetation types (Appendix S3), it appears that a mixture of grassland/moss/CWD and young trees/bushes, within a predominantly mature native forest matrix is required for the species’ survival. Short vegetation increases water retention while decreasing temperature at the soil level and provides a good refuge from predators [50], [67]. The microhabitats of all monitored populations showed >70% relative humidity and <22°C air temperature (Appendix S3). These data were all obtained in the daytime during the Austral spring-summer and, therefore, most likely represent values near the minimum humidity and maximum temperature requirements for the species. Studies on habitat selection of Darwin’s frogs are required to better understand the impacts of habitat loss as well as to develop adequate conservation management practices [67].

### Abundance of Rhinoderma Darwinii

Capture-mark-recapture analysis indicated considerable variability in detection probability between sites (0.04–0.64 per site visit), suggesting that a degree of caution is needed in interpreting the RAI values. Nonetheless, the magnitude of RAI differences between sites is large enough to give some confidence that the broad pattern of higher abundance on Chiloé Island than elsewhere is real, particularly for the eight sites located in the southern third of the island. It is probably not coincidental that human perturbation is minimal in this area, much of which is protected.

As *R. darwinii* exhibits a high degree of site fidelity [9], the use of non-invasive closed population CMR analysis, appears to be a reliable method to estimate population size in this species. There was, however, strong support for heterogeneity and behaviour effects on detection probability at the one site where there were many (16) visits, but not at sites with fewer (4) visits. This is likely because of insufficient data at the less-intensively visited sites to identify these effects in capture probability, suggesting that abundances at these sites could had been under-estimated to some extent. Nonetheless, any such underestimation is likely to be modest, since the estimated degree of heterogeneity in capture probability was not great. Furthermore, the sites at which CMR analysis was applied included both large and small populations, as indicated by RAI values. Taken together, these lines of evidence suggest that all of the sites visited have population sizes below 100, giving a picture of highly fragmented, small and vulnerable populations.

### Conservation Status

#### Rhinoderma rufum

More than three decades has passed since *R. rufum* was last detected [20]. This species’ habitat, the Maulino forest, is today scarcely represented within the Coastal range of central-south Chile [24]. Although there are no objective records about the presence of this species within WPAs, there are eight National Reserves and one National Park within its historical range. However, most of these are small (45–9,262 ha), have been explored in detail by park rangers, herpetologists and other scientists and are frequently visited by tourists. In this respect, it is important to note that *Rhinoderma* spp. is not the hardest species to find and identify, particularly during their reproductive season, since they have a: 1) unique anatomy; 2) characteristic call (both sexes); 3) diurnal activity; and 4) gregarious behaviour. Given the size of its historical distribution, hope remains that *R. rufum* still survives somewhere to this day [43]. Therefore, we suggest the species should be considered as a candidate for the new IUCN Red List category Critically Endangered (Possibly Extinct; CR [PE]), currently under review by the IUCN [68].

#### Rhinoderma darwinii

Although *R. darwinii* abundance has not been previously recorded, and hence the extent of population declines cannot be accurately measured, our results indicate that there has been a recent rapid population decline of this species, based on the severe reduction in distribution range, anecdotal data on severe population declines (*e.g.* at Melimoyu) and local extinctions registered within WPAs, remote areas and undisturbed ecosystems. Alarmingly, the causes of these declines are not understood. Even though the species remains widely distributed, mainland populations are small and highly fragmented. Following the A2abc IUCN Red List criteria, our new data suggest this species should be re-classified at least as Endangered (EN). Should further work show the observed decline to be greater than 80% over 10 years, then the species would qualify for a listing of Critically Endangered (CR).

### Conclusions

The current situation with *R. rufum* and *R. darwinii* is disconcertingly similar to the case of the Northern and Southern gastric brooding frogs (*Rheobatrachus vitelinus* and *R. silus*) which disappeared from the rain forests of Eastern Australia. Like the mouth brooding Darwin’s frogs, there were just two species of gastric brooding frogs which experienced rapid and enigmatic declines to extinction, suspected to have been caused by a series of factors, including amphibian chytridiomycosis [69]. Their phenomenal reproductive strategy disappeared over twenty years ago with the extinction of both species and may never evolve again.

Although additional studies are needed in order to fill gaps in the knowledge of *Rhinoderma* spp., our study provides the best evidence yet on the extent of declines and conservation status of Darwin’s frogs. Despite extensive searches, we were not successful in finding *R. rufum*. Once abundant, today there is a high probability that this species is extinct. Tagging the Critically Endangered *R. rufum* as Possibly Extinct (PE) may help to focus conservation efforts on its sister species, *R. darwinii*. Although it has a current range covering a vast area of south Chile and Argentina, *R. darwinii* occupies only a tiny percentage of this area as small, fragmented populations. Our data suggest *R. darwinii* should be reclassified as Endangered.

## Supporting Information

Appendix S1 **Examined archived** ***Rhinoderma*** **spp.**
(PDF)

Appendix S2 **Historical sightings of** ***Rhinoderma rufum*** **.**
(PDF)

Appendix S3 **Habitat characteristics of extant** ***Rhinoderma darwinii*** **populations.**
(PDF)
