# Supplementary material for: The Population Decline and Extinction of Darwin’s Frogs
Source: PLoS One. 2013 Jun 12;8(6):e66957. doi: 10.1371/journal.pone.0066957 (PMC3680453; doi:10.1371/journal.pone.0066957)
Supplement: Appendix S1 — Examined archived Rhinoderma spp. (PDF) [file pone.0066957.s001.pdf]

S1. Examined archived post-metamorphic specimens of Darwin's frogs (*Rhinoderma darwinii* and *Rhinoderma rufum*), for which information on the date and location of collection were obtained.

Institution	Standard Symbolic Code[a]	Country of institution	*R. darwinii*	*R. rufum* (number newly identified[b])
Field Museum Natural History, Chicago.	FMNH	USA	102	428 (428)
Zoologisches Museum Hamburg.	ZMH	Germany	100	221 (41)
Naturhistorisches Museum, Wien.	NHMW	Austria	193	1 (1)
Royal Belgian Institute of Natural Sciences.	RBINS	Belgium	17	118 (118)
Museo de Zoología, U. de Concepción.	MZUC	Chile	112	9 (0)
University of Kansas Natural History Museum.	KU	USA	85	0
Museum of Comparative Zoology, Harvard Univ.	MCZ	USA	40	29 (29)
Texas Cooperative Wildlife Collection, Texas A&M Univ.	TCWC	USA	0	65 (65)
Centro de Investigaciones Zoológicas, U. de Chile.	CIZ	Chile	45	16 (0)
Zoologisches Forschungsmuseum Alexander Koenig.	ZFMK	Germany	49	9 (3)
Zoologische Staatssammlung München.	ZSM	Germany	55	3 (0)
Museo Argentino Cs. Naturales Bernardino Rivadavia.	MACN	Argentina	53	0
Carnegie Museum of Natural History, Pittsburgh.	CM	USA	7	40 (40)
Natural History Museum, London.	BMNH	UK	29	11 (11)
Fundación Miguel Lillo, Tucumán	FML	Argentina	39	0
Muséum National d'Histoire Naturelle, Paris.	MNHN	France	24	12 (10)
Florida Museum of Natural History, Univ. of Florida.	FLMNH	USA	32	0
Nat. Museum Natural History, Smithsonian Institution.	USNM	USA	21	10 (8)
American Museum of Natural History, New York.	AMNH	USA	6	21 (21)
Michigan State University Museum.	MSUM	USA	24	0
University of Michigan Museum of Zoology.	UMMZ	USA	17	5 (0)
Museum für Naturkunde der Humboldt-Univ. Berlin.	ZMB	Germany	22	0
Museum of Vertebrate Zool., U. California at Berkeley.	MVZ	USA	18	0
California Academy of Sciences.	CAS	USA	15	0

Lousiana Museum of Natural History.	LSUMZ	USA	14	1 (1)
Museo Nacional de Historia Natural, Santiago.	MNHNC	Chile	14	0
Naturhistoriska Riksmuseet, Stockholm.	NRM	Sweden	10	0
Museo de Ciencias Naturales, Madrid.	MNCN	Spain	9	0
Senckenberg Naturmuseum, Frankfurt.	SMF	Germany	7	2 (2)
Texas Natural History Collections, U. of Texas at Austin.	TNHC	USA	9	0
Fac. Cs. Naturales y Museo, U. Nacional de la Plata.	-	Argentina	7	0
National Museum, Prague.	MNHP	Czec Rep.	7	0
Natural History Museum of Los Angeles County.	LACM	USA	4	3 (0)
Zoologiska Museet, Lunds Universitet.	ZMUL	Sweden	6	0
Hungarian Natural History Museum.	MNHM	Hungary	5	0
Senckenberg Naturmuseum, Dresden.	MT D	Germany	5	0
Staatliches Museum für Naturkunde, Stuttgart.	SMNS	Germany	5	0
University Museum of Zoology, U. of Cambridge.	UMZC	UK	1	4 (4)
Academy of Natural Sciences, Philadelphia.	ANSP	USA	0	4 (4)
Estación Biológica Doñana.	EBD-CSIC	Spain	4	0
Yale University, Peabody Museum of Natural History.	YPM	USA	3	0
Museo Regionale di Scienze Naturali, Torino.	MRSN	Italy	0	3 (0)
Cornell University Museum of Vertebrates, Ithaca.	CU	USA	3	0
U. Louisiana at Monroe, Museum of Natural History.	NLU	USA	3	0
Zoologisches Institut, Universität Wien.	ZIUW	Austria	0	3 (3)
University of Texas at Arlington.	UTA	USA	1	0
Utah Museum of Natural History, University of Utah.	UMNH	USA	1	0
Zoological Museum Amsterdam, U. of Amsterdam.	ZMA	Netherlands	1	0
Arizona State University.	ASU	USA	1	0
Museo del Mar, Universidad Arturo Prat.	MUAP	Chile	1	0
**TOTAL**			**1,226**	**1,018**

[a]According to Sabaj (2010).

[b]Specimens previously identified as *Rhinoderma darwinii* but determined to be *Rhinoderma rufum* in the current study

[illegible]
