# Supplementary material for: The Population Decline and Extinction of Darwin’s Frogs
Source: PLoS One. 2013 Jun 12;8(6):e66957. doi: 10.1371/journal.pone.0066957 (PMC3680453; doi:10.1371/journal.pone.0066957)
Supplement: Appendix S2 — Historical sightings of Rhinoderma rufum . (PDF) [file pone.0066957.s002.pdf]

S2. Historical sightings by year of the Northern Darwin's frog (*Rhinoderma rufum*) based on museum archives and the scientific literature.

Year	Locality	Collector or observer	Reference
1861-2	Vichuquén	L. Landbeck.	Philippi 1902.
1903	Hualqui	C.S. Reed.	ANSP 15884-7.
1904	Arauco/Valdivia?	C.S. Reed.	BMNH 1904.10.26.109-10/111-16.
1908	Los Quillayes/Hualañé/ Concepción	R. Barros/T. Barbour.	Barros 1918/UMMZ 50109.
1909	Concepción	T. Barbour.	UMZC R.15590.
1911	Valdivia?	C.E. Porter.	BMNH 1911.3.13.4.
1912	Ranguilí	R. Barros .	Barros 1918
1921	No data	Capt. Dollman.	BMNH 1921.2.18.1-2.
1922	Concepción/Panguipulli?	Carlos Reed.	MCZ A8548-60.
1926	Santiago?	Timmerman.	ZFMK 8340-49.
1927	Cerro Caracol	O. Wilhelm.	Wilhelm 1927
1929	Concepción	P. Ralun.	ZMH A10935-6.
1930	Valparaiso/Concepción	D. Kuhlmann/O. Wilhelm.	ZSM 1838-40/ZIUW Q169.
1931	Concepción	P. Ralun.	ZMH A10957-74.
1938	Nueva Aldea	J. Theune.	ZMH A10975-95.
1951	Barranca Alta	D. Torres.	Torres & Castillo 1973
1957	Chiguayante	J. Cei.	Cei 1962
1962	Concepción	R. Donoso-Barros.	MZUC 11071, 24833-40.
1966	Zapallar	C. Moreno.	Formas et al. 1975.
1969	Vichuquén	A. Morán & G. Tapia.	MNHN 1978.253
1970	Contulmo?	T. Cekalovic.	FMNH 200901-2.
1973	Chiguayante/Concepción	J. Pugin, A. Moran & G.	MHNH 1978.252/USNM 218050.

		Tapia.	
1974	Chiguayante	No data.	USNM 218049.
1975	Chiguayante/Río Longaví	T Cekalovic/E. Vilo.	FMNH 202256-325/ZMH A04439-613/TCWC 53842-905/RBINS 11344-47, 49-52, 11368/G. Medina-Vogel pers. comm.
1976	Chiguayante	E. Vilo.	RBINS 11348/FMNH 207501-80, 209393-425.
1977	Paredones/Hualqui/ Chiguayante	J. Gutierrez/E. Vilo	CIZ 112/FMNH 211071-141.
1978	Concepción	No data.	LACM 164895.
1979	Chiguayante	T. Cekalovic	FMNH 209292-391, 211154-209.
1980	San Pedro	M. Penna & A. Veloso.	Penna & Veloso 1990.
