# Supplementary material for: The Population Decline and Extinction of Darwin’s Frogs
Source: PLoS One. 2013 Jun 12;8(6):e66957. doi: 10.1371/journal.pone.0066957 (PMC3680453; doi:10.1371/journal.pone.0066957)
Supplement: Appendix S3 — Habitat characteristics of extant Rhinoderma darwinii populations. (PDF) [file pone.0066957.s003.pdf]

S3. Summary of habitat description for 36 extant populations of the Southern Darwin's frog (*Rhinoderma darwinii*).

Area	Population	Within WPA	Human use and impact*	Temp (°C)	Humidity (%)	Vegetation coverage (%)		
						F[a]	B/S[b]	G/M/CWD[c]
Coast	Butamalal	no	+	13.7	75.8	80	15	5
	El Natre 1	no	+	19.9	91.2	70	10	20
	El Natre 2	no	+	16.4	84.9	40	30	30
	RN Contulmo	yes	+	12.9	89.3	85	10	5
	RF Contulmo	no	–	15.7	82.8	70	15	15
	Oncol	yes	–	9.8	89.9	95	5	0
	Alerce Costero	yes	+	17.3	80.4	40	30	30
Andes	Huerquehue	yes	+	15.5	78.4	25	25	50
	Villarrica 1	yes	–	12.3	84.7	10	80	10
	Villarrica 2	yes	–	14.9	74.2	40	40	20
	Coñaripe	no	+	13.3	86,2	70	10	20
	Puma	yes	–	16.3	73.2	90	10	0
	S. Botánico	yes	+	21.7	79.3	50	25	25
	Huilo-Huilo	yes	+	15.1	80.1	80	10	10
	Pudu	yes	+	19.0	86.1	80	10	10
	Puyehue 1	yes	+	14.1	93.4	85	10	5
	Puyehue 2	yes	+	14.8	89.8	95	5	0
	Pajaritos	yes	+	15.5	86.1	95	0	5
Chiloé	S. Darwin 1	yes	+	13.3	92.1	80	15	5

	S. Darwin 2	yes	+	13.4	99.9	80	20	0
	Alerzales	no	+	15.4	89.3	85	15	0
	Yaldad	Yes	+	13.9	95.6	30	35	35
	Cerro Mirador	Yes	–	16.1	80.9	30	60	10
	Huillín	yes	–	15.8	81.4	30	60	10
	Mirador	yes	–	11.4	81.4	10	90	0
	Anteinío	yes	–	11.0	89.1	70	10	20
	Inío 1	yes	–	13.9	90.1	60	25	15
	Inío 2	yes	–	11.2	92.1	50	40	10
	Faro	yes	+	17.0	79.0	80	10	10
South	Melimoyu 1	no	++	14.0	75.5	0	10	90
	Melimoyu 2	no	++	18.8	73.2	60	20	20
	Queulat 1	yes	++	15.8	85.6	25	50	25
	Queulat 2	yes	+	16.0	85.0	40	50	10
	Queulat 3	yes	+	16.5	74.6	80	15	5
	Queulat 4	yes	+	15.5	85.1	85	10	5
	Queulat 5	yes	+	17.3	75.0	10	70	20
**AVERAGE**				**15.1**	**84.2**	**59**	**26**	**15**

* – = none, population whithin in a wild protected area (WPA) or undisturbed ecosystem; + = low, population in a exploited native forest for firewood or near a trail path frequently transited within a WPA; ++ = high, population in a severely exploited native forest, or near a town or development infraestructure.

[a] Forest; [b] Shrub and bushes; and [c] Grassland, moss and coarse woody debris.
